# The Influence of Maxillary Incisor Labiolingual Inclination on Smiling Profile Esthetics Among Saudis

**DOI:** 10.7759/cureus.20966

**Published:** 2022-01-05

**Authors:** Mohammed Albwardi, Saad Albwardi, Khalid Dobaian, Khalid Alqahtani, Abdulaziz Altayir, Abdulaziz Almutawa

**Affiliations:** 1 Orthodontics and Dentofacial Orthopedics, Riyadh Elm University, Riyadh, SAU; 2 General Dentistry, Riyadh Elm University, Riyadh, SAU

**Keywords:** smile, incisors, esthetic, inclination, digital orthodontics

## Abstract

Objectives

The study aimed to (1) determine the impact of maxillary incisor inclination on profile view esthetic perception, (2) determine the most esthetic inclination and correlate it with a profile view, and (3) evaluate the difference in the perception of orthodontists, dentists, and laypeople toward incisor inclination attractiveness in Saudi Arabia.

Materials and methods

A well-balanced smiling photograph of a male adult who fulfilled the criteria for soft tissue and cephalometric values was taken from a profile view. The photograph was modified to stimulate three inclinations toward the labial and palatal side each. The most retroclined photograph showed −15° inclination, and the most proclined photograph showed +15° inclination, with the originally taken photograph being neutral at 0° inclination. Thus, we obtained seven photographs with 5° of difference between each. All photographs were randomly distributed in a questionnaire form filled by 135 participants.

Conclusion

The most attractive reported inclination was −5° inclination, while the least attractive inclination was +15° inclination. Excessive proclination has been less desirable than retroclination. The profile smiling view is very useful in evaluating the inclination of the labial face tangent and should be considered a standard view for orthodontic photographic records.

## Introduction

A smile is a very important facial characteristic, being a very frequent facial expression. When teeth are well organized and aligned in their natural anatomic position, a smile could be an attractive feature itself, having a positive impact on a person’s daily life. Regarding the anatomic position of teeth, an agreement exists between orthodontists and dentists on the majority of general anatomic features and teeth position, although some details, such as the labiolingual inclination of maxillary incisors, are still disputed in literature and clinical practice. Attention to such details is important as patients tend to be more esthetically demanding of certain dental features, considering the influence that media can have on esthetic standards [1]. Geron et al. demonstrated that females are particularly expected to have more oral and dental attractiveness than males [2]. Moreover, successful orthodontic treatment does not necessarily ensure an attractive smile, even if it meets the standards of the American Board of Orthodontics [3]. Both frontal and lateral views are significantly important when assessing orthodontic treatment [4]. The inclination of maxillary incisors is an important profile view component, although a dispute regarding the decision on the most esthetic inclination exists in the literature as some studies are contradictory. De Velasco et al. indicated that people preferred either upright or slightly proclined incisors [5]. Other studies mentioned that a protrusive maxillary dentition is better to be left in its natural upright position instead of retracting the anterior maxillary teeth, assuming that retroclination is a characteristic of older people’s appearance due to loss of torque [6]. On the other hand, Doshi et al. indicated that incisors are preferred to be either upright or slightly retroclined [7]. Cao et al. also indicated that incisors are preferred to be upright or slightly retroclined, and it was mentioned that proclination could ruin an esthetically pleasing smile [1]. To resolve this dispute, at least among the Saudi population, it is important to conduct a similar study. Therefore, this study had three major purposes: (1) to determine the impact of maxillary incisor inclination on the esthetic profile view perception, (2) to determine the most esthetic inclination and correlate it with the facial features of a profile view, and (3) to evaluate the difference in the perception between orthodontists, dentists, and laypeople toward the attractiveness of incisor inclination in Saudi Arabia.

## Materials and methods

Subject

A 28-year-old male was chosen for the study from a private clinic in Riyadh. A signed informed consent form was obtained from the participant, and ethical approval was obtained from the research center at Riyadh Elm University. The clinical and lateral cephalometric examinations showed that he met the following criteria: (1) harmonious smile in both frontal and profile views with normal nasolabial and labiomental folds, (2) class I canine and molar relationship with normal overjet and overbite, (3) well-positioned maxillary incisors according to cephalometric standards, and (4) normal profilometric measurements. The right lateral photo of the patient was taken using a Nikon camera and Sigma lenses with a flash ring while the patient was fully smiling in a natural head position. The same photo was used for computer-aided modifications. The use of computer-aided modification proved to be a promising method for research on patients’ and dental professionals’ preferences in esthetic dentistry [8]. With the use of the right photographic tools, knowledge about the photographic techniques, and the aid of software tools, such as Photoshop, dentists and dental practitioners can treat cases in more sophisticated ways. Understanding these tools and techniques will also prove to be invaluable for communications between dentists and the laboratory, other specialists, and patients [9].

Image modification

**Figure 1:**
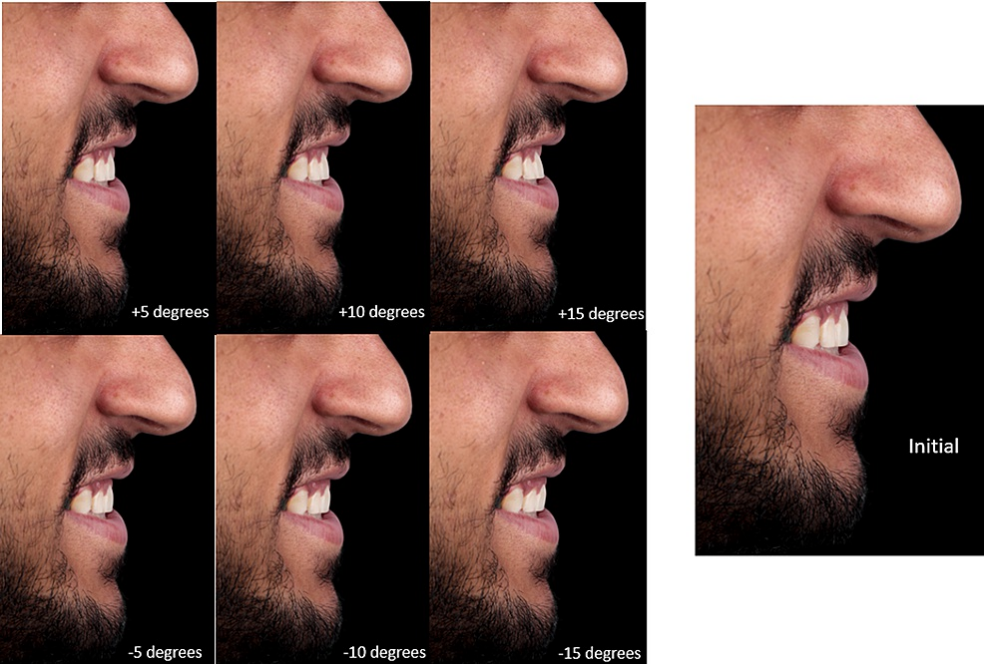
The original photograph of the subject is labeled “initial,” and the six modifications are produced at the labial and palatal directions, each labeled with the adjusted degree of rotation.

**Figure 2:**
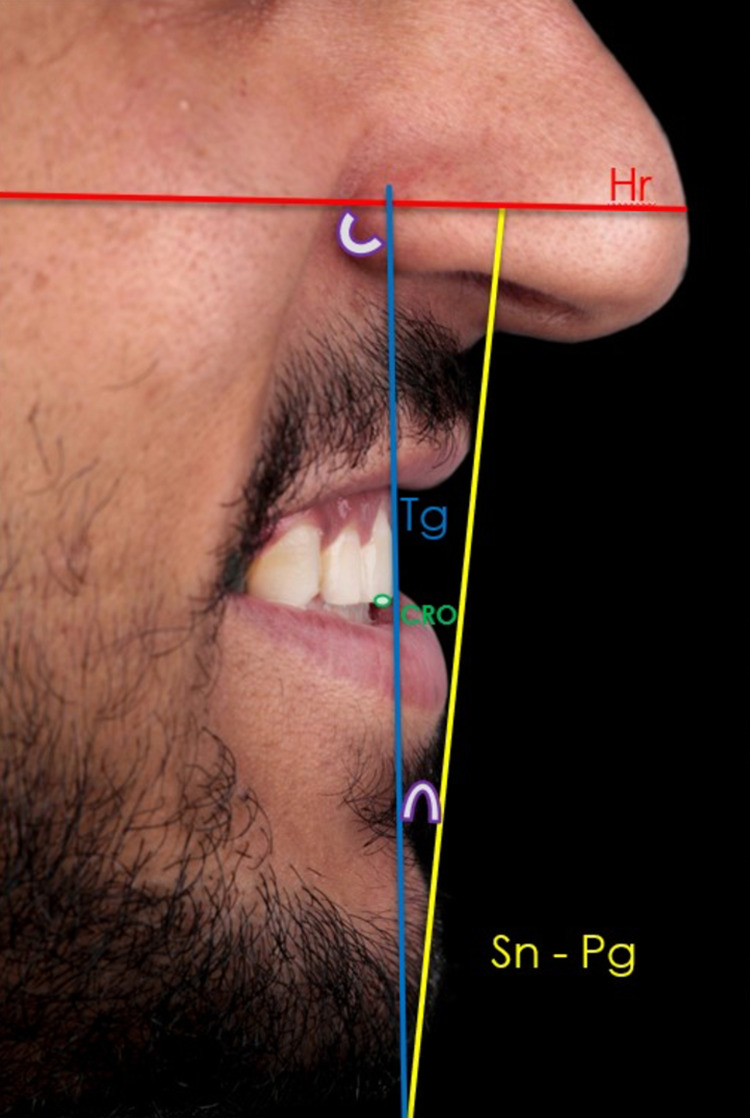
Angular measurements of incisor inclination on the original profile photograph. Hr: horizontal line, Tg: tangent to the labial surface of the maxillary central incisor, Sn–Pg′: line connecting the subnasal point (Sn) (the deepest point on the curve where the profile of the nose joins the lip) to facial pogonion (Pg′), CRO: pointed at the midpoint of the mesiodistal width of the incisal edge to achieve symmetry.

The obtained photograph required delicate editing using a software program (Adobe Photoshop CC 2015) suitable for it (Figure 1). According to the method described by Ghaleb et al., only the angle between the horizontal line (Hr) and tangent (Tg) was edited (Table 1) [4]. Crowns of both central and lateral incisors were cut using the cutting tool of the program. Each tooth that was edited was considered as an object, which necessitated having a center of rotation (CRO), which was the incisal edge in this case. Regarding the central incisor, the incisal tip traced in the lateral cephalogram was the CRO. As for the lateral incisor, to achieve symmetry, the CRO had to be at the midpoint of the mesiodistal width of the incisal edge (Figure 2). Horizontal tangents for both central and lateral incisors were at the level of incisal edge to conserve vertical positions. The lateral limit for the sagittal repositioning of the lateral incisors was the vertical tangent medial to the canine teeth. All simulations were in 5° increments starting from the initial point, which is considered to be 0°. Three modifications were directed labially, and three modifications were directed palatally (Figure 1). Artistic editing was undertaken when necessary to maintain a natural appearance. Seven final images were obtained and uploaded in a Google Form with a random arrangement.

**Table 1:** Angular measurements of incisor inclination of the face in the seven photographs. Hr: horizontal line, Tg: tangent to the labial surface of the maxillary central incisor.

Photograph	Angle Tg/Hr (°)
-15°	76
-10°	81
-5°	86
Initial	91
+5°	96
+10°	101
+15°	106

Judges

To judge the profile photographs, three panels were formed with a total of 135 participants: 45 dentists, 45 orthodontists, and 45 laypeople. The dentists and orthodontists had completed their professional training. The laypeople consisted of adults (more than 18 years old).

Incisor inclinations

To help the orthodontists determine the most esthetic position for maxillary incisor inclination, the profile view photograph was taken while the head was in the esthetic position as suggested by Bass (2003) [10]. The horizontal line (Hr) is an esthetic line that is not changed by treatment. It acts as a reference line for the chin position if it has been adjusted by an orthopedic or orthognathic intervention. The Sn-Pg′ line, joining the subnasal point (Sn) (the deepest point on the curve where the profile of the nose joins the lip) to facial pogonion (Pg), represents the lower facial third, i.e., the nearest reference part of the face to the incisors.

For each photograph, the following was done to obtain angular measurements:

1. An Sn-Pg′ line and an Hr line passing through the mid-third were drawn.

2. The most anterior point of the labial surface of the maxillary incisor was determined, which was obtained by the intersection of the surface with the vertical tangent.

3. Tg′ was drawn passing through this point.

Two angular measurements for each inclination were obtained (Figure 2):

1) Tg/Hr′: the angle between the horizontal line and the incisor inclination.

2) Tg/Sn-Pg′: the angle between the lower facial third and the incisor inclination; this particular angle has a positive value when the tangent is forward and a negative value when the tangent is backward.

Evaluation of incisor inclination

The judges were provided with instructions on the scale use (very not attractive, not attractive, average, attractive, and very attractive) and chose one answer for each photograph. No information was provided regarding the faces they were to see other than being told that the subject was a male. The judges viewed all the photographs, which were arranged randomly. The arrangement of the images was as follows: image A (+15°), image B (−15°), image C (+5°), image D (−5°), image E (+10°), image F (−10°), and image G (initial, 0°). Instructions were given to all 135 judges. The questionnaire was displayed using an Apple iPad Pro 9.7 (2016).

## Results

Statistical analysis

The study sample consisted of 135 participants. The data analysis included two stages: (I) hypothesis testing using the chi-square test, which was performed using IBM SPSS Statistics 25.0, and (II) five-point Likert scale analysis to measure the level of preference of the entire sample without distinction.

The 135 participants were divided into three separate panels, categorized according to professions: orthodontists, dentists, and laypeople. Each panel consisted of 45 participants. Of all participants, 54.8% were males. The participants were adults aged 18-55 years, of whom 41.5% were aged between 25 and 34 years. All participants were Saudi nationals (Table 2).

**Table 2:** Judges’ demographics. All values are presented as numbers and percentages.

Characteristic	n (%)
Nationality (n = 135)	
Saudi	135 (100%)
Non-Saudi	0 (0%)
Gender (n = 135)	
Male	74 (54.8%)
Female	61 (45.2%)
Age (n = 135)	
18–24	26 (19.3%)
25–34	56 (41.5%)
35–55	48 (35.6%)
More than 55	5 (3.7%)
Group (n = 135)	
Dentist	45 (33.3%)
Orthodontist	45 (33.3%)
Other	45 (33.3%)

**Figure 3:**
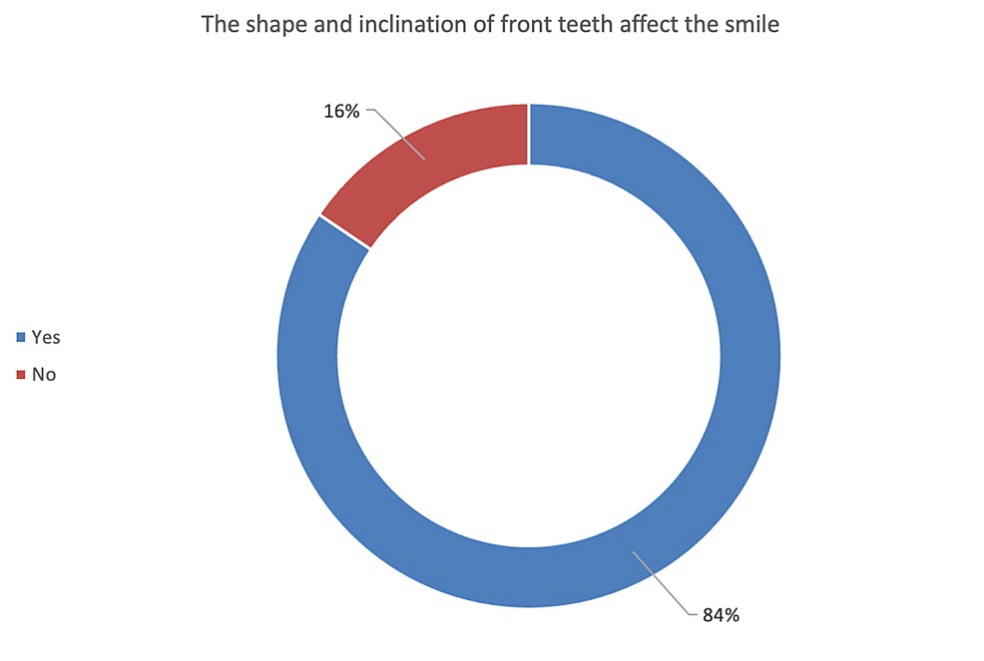
Percentage of people who believe and do not believe that inclination affects the smile.

The participants were asked at the beginning of the questionnaire whether the shape and inclination of frontal teeth would affect the view of a smile (Figure 3), with 84% of the participants answering “yes” and believing that the shape and inclination have an effect, and 16% of participants answering “no” and believing that the shape and inclination do not have an effect (Table 3).

**Table 3:** Judges’ answers whether the shape and inclination of front teeth affect the smile. All values are presented as numbers and percentages.

Variable	n (%)
Yes	114 (84.4%)
No	21 (15.6%)

Regarding the least preferred smile view, all three panels believed that image A (+15° inclination) was the most unattractive, with 26.7% of the dentists, 42.2% of the orthodontists, and 31.1% of the laypeople perceiving it as very not attractive (p = 0.003). Image G (initial, 0° inclination) was the most preferred smile view for orthodontists, as 33% perceived it as very attractive (p < 0.01). Dentists preferred image D (−5° inclination), with 26.7% of them perceiving it as very attractive (p = 0.047), and 20% of the laypeople perceived image F as very attractive (p = 1.04), while 44% of them perceived image D (−5° inclination) as attractive, which was the second to the very attractive option (Table 4).

**Table 4:** Comparison between dentist, orthodontist, and laypeople. Of the dentists, 27% prefer image D, 33% of the orthodontists prefer image G, and 20% of the laypeople prefer image F. Dentists, orthodontists, and laypeople consider image A as very not attractive. Statistically significant at 0.05.

Variable	Dentist (n = 45)	Orthodontist (n = 45)	Laypeople (n = 45)	P-value
	n (%)	n (%)	n (%)	
Picture A (+15°)				
Very not attractive	12 (26.7%)	19 (42.2%)	14 (31.1%)	0.003
Not attractive	17 (37.8%)	22 (48.9%)	12 (26.7%)	
Average	9 (20%)	4 (8.9%)	15 (33.3%)	
Attractive	5 (11.1%)	0 (0%)	4 (8.9%)	
Very attractive	2 (4.4%)	0 (0%)	0 (0%)	
Picture B (−15°)				
Very not attractive	2 (4.4%)	1 (2.2%)	1 (2.2%)	0.009
Not attractive	13 (28.9%)	20 (44.4%)	8 (17.8%)	
Average	22 (48.9%)	19 (42.2%)	16 (35.6%)	
Attractive	6 (13.3%)	5 (11.1%)	15 (33.3%)	
Very attractive	2 (4.4%)	0 (0%)	5 (11.1%)	
Picture C (+5°)				
Very not attractive	0 (0%)	0 (0%)	2 (4.4%)	0.262
Not attractive	8 (17.8%)	9 (20%)	13 (28.9%)	
Average	26 (57.8%)	23 (51.1%)	20 (44.4%)	
Attractive	7 (15.6%)	12 (26.7%)	8 (17.8%)	
Very attractive	4 (8.9%)	1 (2.2%)	2 (4.4%)	
Picture D (−5°)				
Very not attractive	1 (2.2%)	0 (0%)	3 (6.7%)	0.047
Not attractive	4 (8.9%)	4 (8.9%)	5 (11.1%)	
Average	19 (42.2%)	17 (37.8%)	11 (24.4%)	
Attractive	9 (20%)	10 (22.2%)	20 (44.4%)	
Very attractive	12 (26.7%)	14 (31.1%)	6 (13.3%)	
Picture E (+10°)				
Very not attractive	0 (0%)	2 (4.4%)	8 (17.8%)	0.004
Not attractive	22 (48.9%)	14 (31.1%)	14 (31.1%)	
Average	20 (44.4%)	23 (51.1%)	13 (28.9%)	
Attractive	3 (6.7%)	6 (13.3%)	9 (20 %)	
Very attractive	0 (0%)	0 (0%)	1 (2.2%)	
Picture F (−10°)				
Very not attractive	0 (0%)	3 (6.7%)	2 (4.4%)	0.104
Not attractive	8 (17.8%)	5 (11.1%)	6 (13.3%)	
Average	25 (55.6%)	19 (42.2%)	14 (31.1%)	
Attractive	9 (20%)	14 (31.1%)	14 (31.1%)	
Very attractive	3 (6.7%)	4 (8.9%)	9 (20%)	
Picture G (0°)				
Very not attractive	0 (0%)	0 (0%)	1 (2.2%)	0.000
Not attractive	6 (13.3%)	1 (2.2%)	10 (22.2%)	
Average	19 (42.2%)	9 (20%)	19 (42.2%)	
Attractive	18 (40%)	20 (44.4%)	13 (28.9%)	
Very attractive	2 (4.4%)	15 (33.3%)	2 (4.4%)	

**Figure 4:**
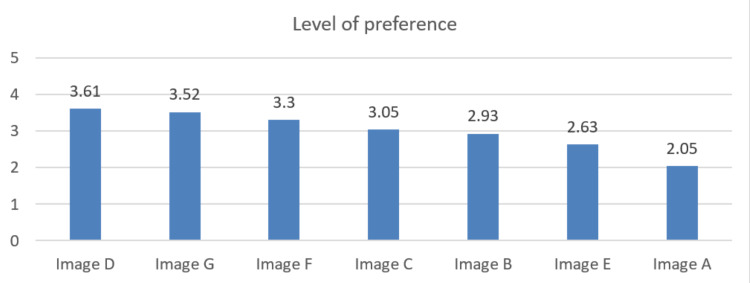
Level of preference toward the pictures (five-point Likert scale analysis).

A Likert scale analysis was done to measure the appreciation of the images from all groups without distinction (Figure 4). The choice with the highest score was very attractive (range: 5-4.21), and the lowest score choice was very not attractive (range: 1.8-1) (Table 5). The most highly appreciated image was image D (−5° inclination), scoring 3.61 on the five-point Likert scale. Second to image D (−5° inclination) was image G (initial, inclination 0°), scoring 3.52. Image F (−10° inclination) was the third, scoring 3.3. The least appreciated image was image A (+15° inclination), scoring 2.05.

**Table 5:** The five-point Likert scale given weight.

Rate	Verbal interpretation	Range
5	Very attractive	4.21–5.00
4	Attractive	3.41–4.20
3	Average	2.61–3.40
2	Not attractive	1.81–2.60
1	Very not attractive	1.00–1.80

The highest appreciated images on the Likert scale, i.e., images D, G, and F, were correspondent with the images perceived as very attractive by each profession on a chi-square test. Also, the least appreciated image A was the same image perceived as very not attractive by all professions (Table 6).

**Table 6:** Level of preference toward the pictures (five-point Likert scale analysis).

Image	Mean	SD	Rank	Level of preference
Image A (+15°)	2.05	0.972	7	Not attractive
Image B (−15°)	2.93	0.908	5	Average
Image C (+5°)	3.05	0.831	4	Average
Image D (−5°)	3.61	1.045	1	Attractive
Image E (+10°)	2.63	0.835	6	Average
Image F (−10°)	3.30	0.978	3	Average
Image G (0°)	3.52	0.913	2	Attractive

## Discussion

Smile is an essential part of facial esthetics and is essential in most orthodontic treatments. In our study, we focused on how different groups in society perceive inclination, which can be an important treatment outcome. Regarding the anatomic position of teeth, an agreement exists between orthodontists and dentists on most of the general anatomic characteristics and position of teeth. However, some details, such as the labiolingual inclination of maxillary incisors, are still disputed in the literature and clinical practice. Cephalometric values should be a guide for orthodontic treatment, but not its primary goal. Different populations have different preferences on whether maxillary incisors should be proclined, retroclined, or in a neutral position. The gingival display can also affect the esthetics of a smile and influence the treatment result [2]. The first question in this survey was developed to understand the importance of frontal teeth inclination to the three different panels. The vast majority (84%) of the sample agreed that the shape and inclination of frontal teeth do affect smile. This indicates the importance of inclination and awareness to this factor among the three different panels. The least preferred inclination for all panels was +15° in image A. A similar study was conducted in Saudi Arabia by Almutairi et al., who concluded that laypeople and dental professionals in Riyadh, Saudi Arabia, seem to disfavor the proclination of frontal teeth despite the subjects having a class I molar relationship [11]. A chi-square test was done to understand the preferred inclination for each group, and it was concluded that orthodontists preferred the initial inclination of 0°, dentists preferred the inclination of −5°, and laypeople preferred the inclination of −10°. A Likert scale analysis was done to conclude which inclination was preferred by all panels combined, showing that the highest three scoring inclinations were −5°, initial 0°, and −10° inclination, which was parallel with the panels’ most preferred images. The same finding can be noted for the least preferred image by all panels, the inclination of +15°, which had the lowest score. The highest scoring inclination was of −5° in image D, making it the most preferred inclination viewed by the sample in this study, while the inclination of +15° in image A had the lowest score in consensus with the chi-square test, making it the least preferred inclination. This study, in addition to Almutairi et al.’s study, with differences in methodology considered, indicated a tendency to dislike maxillary protrusion and incisor proclination. Aldrees mentioned that the Saudi population is more likely to have a class II facial pattern with a convex profile and more proclined incisors compared to Caucasians [12]. It would be interesting to know whether the prevalence of such features in the population did influence the tendency to dislike incisor proclination and maxillary protrusion. Conducting more studies to investigate the reasons for such tendency is thus recommended.

The limitations of this study comprised various obstacles caused by COVID-19, including social distancing and a limited number of people in a studied area. Inability to standardize the surrounding environment while conducting the interview, such as controlling the amount of light in the room and surrounding noise, was challenging. Additionally, it was not known to what extent the gender of the subject did influence the opinion of this sample. It is recommended to conduct similar studies targeting both genders, both as subjects and as a part of the differences in preference. Considering the patient’s preference in the treatment plan and deciding the final esthetic outcome of dental treatment is highly recommended and important. The use of software editing tools, such as Photoshop, can be very helpful in providing a visual estimation of the final treatment outcome, which can be beneficial in understanding and managing patients’ expectations.

## Conclusions

The most attractive inclination according to all groups was the inclination of −5°, while the least attractive inclination was the inclination of +15°. Excessive proclination has been shown as less desirable than retroclination. The profile smiling view is very useful to evaluate the inclination of the labial face tangent and should be considered a standard view for orthodontic photographic records.
